# The Cardio-Biliary Reflex in Gallbladder Disease: A Case Report and Literature Review

**DOI:** 10.7759/cureus.94272

**Published:** 2025-10-10

**Authors:** Rafat Shehata, Amr Anos, Mohammed Fathy Kandil Mohammed, Mostafa Hekal, Mohamed Elatiky

**Affiliations:** 1 Emergency Medicine, Hampshire Hospitals NHS Foundation Trust, Basingstoke, GBR; 2 Cardiology, Hampshire Hospitals NHS Foundation Trust, Basingstoke, GBR; 3 General Practice, Bolton NHS Foundation Trust, Bolton, GBR; 4 General Practice, Royal Devon University Healthcare NHS Foundation Trust, Barnstaple, GBR

**Keywords:** bradyarrhythmia, cardio-biliary reflex, cholelithiasis, cope’s sign, electrocardiogram (ecg)

## Abstract

This case report describes an elderly female presenting with simultaneous biliary colic and symptomatic sinus bradycardia. The resolution of her bradycardia with pain management alone suggested Cope’s sign, or the cardio-biliary reflex (CBR), as the underlying cause. This reflex is a well-recognized physiological response in which gallbladder pathology triggers vagal nerve signals, potentially causing significant alterations in cardiac rhythm and often mimicking primary heart conditions. This case illustrates that even uncomplicated biliary colic can induce this effect and emphasizes the importance for clinicians to consider this reflex in the differential diagnosis of bradyarrhythmia accompanied by abdominal pain. Recognizing CBR is crucial for directing management toward the correct biliary pathology and avoiding unnecessary cardiac investigations or interventions.

## Introduction

The clinical presentation of acute gallbladder disease, including biliary colic, cholecystitis, and cholelithiasis, typically comprises right upper quadrant pain, fever, nausea, and vomiting. The cardio-biliary reflex (CBR) represents a well-documented yet underrecognized manifestation of acute gallbladder disease, presenting with profound cardiovascular symptoms [1,2]. First described in the early 20th century, this reflex is triggered by afferent stimuli from an inflamed or distended biliary tract, leading to an efferent parasympathetic surge via the vagus nerve [3]. The resulting clinical manifestations include significant bradyarrhythmias, hypotension, syncope, and ECG changes [4,5]. These symptoms often mimic primary cardiac diseases, creating diagnostic challenges and frequently prompting extensive, and sometimes unnecessary, cardiac investigations while the underlying biliary pathology remains undiagnosed [6].

Although most commonly reported in the context of acute cholecystitis, CBR can also be triggered by uncomplicated biliary colic. This scenario is less emphasized in the literature, contributing to its underrecognition in clinical practice [7,8]. Herein, we present an illustrative case of symptomatic sinus bradycardia secondary to uncomplicated biliary colic, which resolved completely with analgesia and conservative management. Through this case and a contemporary review of the literature, we aim to clarify the pathophysiology, expand the clinical spectrum beyond cholecystitis, and reinforce the management principles of this intriguing reflex. We highlight the critical importance of considering CBR in the differential diagnosis of any patient presenting with unexplained bradyarrhythmia concurrent with abdominal pain.

## Case presentation

A 75-year-old woman presented to our hospital with acute epigastric pain radiating to the right upper quadrant, which had begun 24 hours prior to admission. The pain was persistent and unrelieved by paracetamol. She subsequently developed nausea and dizziness.

On initial assessment, she was noted to have bradycardia at 40 beats per minute; the remainder of her vital signs were unremarkable. Abdominal examination revealed a soft abdomen with localized tenderness in the right upper quadrant and epigastrium. There were no signs of peritoneal irritation, and Murphy’s sign was negative. Chest and cardiovascular examinations were normal.

Her medical history was significant for hypercholesterolemia, known cholelithiasis, and elevated body mass index. She had no history of thyroid disease and was not taking any rate-controlling medications.

**Figure 1:**
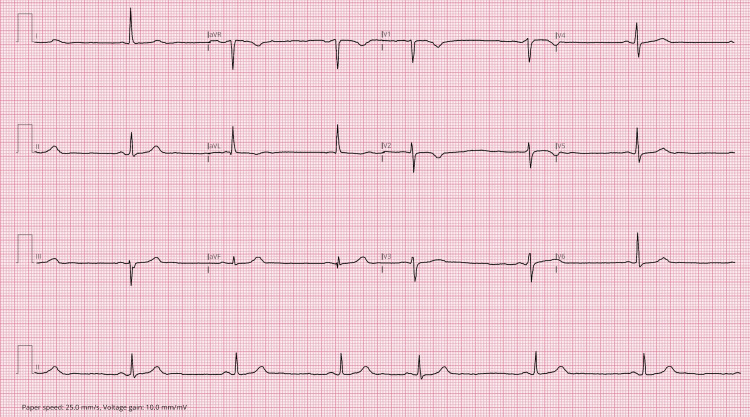
ECG showing sinus bradycardia at 41 bpm with an atrial ectopic beat

Initial laboratory investigations revealed normal inflammatory markers, markedly elevated serum amylase, and deranged liver function tests (Table 1). Thyroid function tests were within normal limits. Electrocardiography confirmed sinus bradycardia at 41 beats per minute, with an atrial ectopic beat (Figure 1).

**Table 1:** Laboratory investigations Initial laboratory results with reference ranges; abnormal values are shown in bold.

Test	Result	Reference range
Serum amylase		
Amylase	2955	28-100 U/L
C-reactive protein	4	0-5 mg/L
Full blood count		
Hemoglobin	145	120-160 g/L
Leucocytes	10	4-11 × 10⁹/L
Platelets	256	150-450 × 10⁹/L
Red blood cell count	4.44	3.8-5.8 × 10¹²/L
Hematocrit	0.41	0.36-0.47 L/L
Neutrophils	8.9	1.5-8 × 10⁹/L
Lymphocytes	0.8	1.3-4 × 10⁹/L
Monocytes	0.3	0.2-1 × 10⁹/L
Eosinophils	0	0-0.8 × 10⁹/L
Basophils	0	0-0.3 × 10⁹/L
Liver function tests		
Bilirubin	20	0-21 µmol/L
Alanine aminotransferase	73	0-35 U/L
Aspartate aminotransferase	101	0-34 U/L
Alkaline phosphatase	106	30-130 U/L
Albumin	41	34-50 g/L
Urea and electrolytes		
Sodium	141	133-146 mmol/L
Potassium	3.8	3.5-5.3 mmol/L
Urea	6.2	2.5-7.8 mmol/L
Creatinine	71	45-84 µmol/L
Estimated glomerular filtration rate	72	60-150 mL/min
Calcium	2.5	2.15-2.6 mmol/L
Corrected calcium	2.49	2.15-2.62 mmol/L
Thyroid function tests		
Thyroid-stimulating hormone	2.12	0.27-4.2 mU/L
Other biochemistry		
Triglyceride	0.89	0-1.7 mmol/L
Lactate dehydrogenase	295	135-214 U/L

Thirty minutes after administration of analgesia, her heart rate improved, and her associated dizziness resolved completely. Abdominal ultrasound revealed a solitary gallstone with no evidence of cholecystitis. A magnetic resonance cholangiopancreatography was initially planned to further evaluate the biliary tree; however, the scan was aborted due to patient claustrophobia.

She was subsequently admitted under the surgical team and managed conservatively with analgesia and intravenous fluids. She was later discharged with a plan for elective laparoscopic cholecystectomy, which was performed successfully several weeks later without immediate postoperative complications or recurrence of bradycardia.

## Discussion

Definition and nomenclature

The term “Cope’s sign” originates from Sir Zachary Cope, who in 1970 described his personal experience with gallbladder disease, initially mistaking the associated epigastric pain and symptoms for cardiac ischemia [1]. Shortly thereafter, O’Reilly and Krauthamer formally reported the association between reflex bradycardia and acute cholecystitis in two patients, coining the term “Cope’s sign” in the medical literature [2]. It is important to distinguish this from the other “Cope’s sign” (psoas test) used in diagnosing appendicitis [5]. The phenomenon is more accurately described as the CBR, a vagally mediated neural arc capable of inducing a spectrum of cardiac disturbances in response to biliary tract pathology [3,6,7].

Pathophysiological mechanism

The accepted mechanism of CBR involves a neural reflex arc. Afferent signals arise from mechanoreceptors and chemoreceptors in the gallbladder wall and biliary tract, stimulated by distension, inflammation, or calculi [8,9]. These impulses ascend via the vagus nerve to the nucleus tractus solitarius in the medulla oblongata, which then sends efferent signals back through vagal cardiac branches, increasing parasympathetic tone. This results in negative chronotropy (bradycardia), dromotropy (atrioventricular conduction delays or blocks), and vasodilation (hypotension) [10,11].

Additionally, the heart and gallbladder share autonomic innervation from spinal levels T4-T6, with intermediate neurons connecting these pathways, facilitating this cross-talk [12]. Animal studies have also demonstrated that gallbladder distension can trigger reflex coronary vasoconstriction, potentially explaining ST-segment changes observed in some cases [9,13,14].

Gallbladder diseases triggering the reflex

CBR is most commonly reported in acute calculous cholecystitis, as documented in numerous case reports [2,4,15]. However, it has also been described in acalculous cholecystitis [16], biliary colic without overt inflammation (as in our case) [17], and even rare entities such as gallbladder torsion [18]. Furthermore, iatrogenic triggers, such as intraoperative gallbladder manipulation, can initiate the reflex arc [19], suggesting that neural irritation, rather than inflammation alone, is the primary stimulus.

Cardiac presentations and ECG changes

The cardiac manifestations of CBR are diverse (Table 2). Sinus bradycardia is the most common presentation, but more severe arrhythmias have been reported, including various degrees of atrioventricular block, from first-degree to complete heart block [6,7,15,17,20-22], as well as sinus pauses or arrest [6,16]. Beyond arrhythmias, chest pain and ECG changes can mimic acute coronary syndrome (ACS), including ST-segment deviations and T-wave inversions, particularly in the inferior leads [13,23-25]. Our patient’s presentation with significant sinus bradycardia falls squarely within this recognized spectrum of rhythm disturbances.

**Table 2:** Summary of existing case reports ABX, antibiotics; AV, atrioventricular; CHB, complete heart block; GB, gallbladder; PM, pacemaker; PPM, permanent pacemaker; RBBB, right bundle branch block; SB, sinus bradycardia; STE, ST elevation

Study	Cases	GB presentation	Cardiac presentation	ECG changes	Complete resolution	Vagolytic	Temporary PM	PPM
O’Reilly and Krauthamer (1971) [2]	2	Acute calculous cholecystitis	Chest pain in Case 1	SB	Cholecystectomy	Atropine in Case 1	No	No
Iftikhar et al. (2022) [3]	1	Acute calculous cholecystitis	Asymptomatic	SB	Conservative Rx	Not reported	No	No
Mainali et al. (2022) [4]	1	Acute calculous cholecystitis	Lightheadedness	SB	Cholecystectomy	Atropine	No	No
Kumar et al. (2020) [6]	1	Acute calculous cholecystitis	Symptomatic bradycardia	Bradycardia with pauses	Analgesia and ABX	No	Yes	No
Franzen et al. (2009) [7]	1	Acute cholecystitis	Syncope	Complete AV block	Cholecystectomy	Not reported	No	No
Meyer et al. (2024) [13]	1	Acute cholecystitis	Chest pain	Inferolateral STE	Cholecystectomy	No	No	No
Patel et al. (2011) [14]	1	Acute calculous cholecystitis	Chest pain	SB + STE	Cholecystectomy	No	No	No
Ola et al. (2020) [15]	1	Acute calculous cholecystitis	Syncope	Complete AV block	Cholecystectomy	No	Yes	No
Lau et al. (2015) [16]	1	Acute acalculous cholecystitis	Not reported	SB/pause	Cholecystectomy	Not reported	No	No
Papakonstantinou et al. (2018) [17]	1	Biliary colic	Asymptomatic	SB + 7 S CHB	Analgesia	No	No	No
Scott et al. (2021) [18]	1	GB torsion	Chest pain & syncope	Bradycardia, No ECG	No	Not reported	No	No
Soric et al. (2015) [20]	1	Acute calculous cholecystitis	Asymptomatic	Intermittent CHB	Cholecystectomy	No	Yes	No
Daliparty et al. (2021) [21]	1	Calculous cholecystitis	Chest pain	Mobitz I AV block	Cholecystectomy	No	No	No
Fang et al. (2021) [22]	1	Cholecystitis and cholangitis	Dyspnea	Complete AV block	Delayed	No	Yes	Yes
Furuhashi et al. (2003) [23]	1	Acute cholecystitis	ECG changes	RBBB + STE	Cholecystectomy	No	No	No
Ozeki et al. (2015) [24]	16	Acute cholecystitis	Chest pain in four cases	Three ST-T ischemic changes	Three surgeries/drains and one ABX	Not reported	No	No
Drachman et al. (2017) [25]	1	Acute cholecystitis	Chest pain	Infero-antero-lateral STE	Surgical drain	No	No	No
Patell et al. (2021) [26]	1	Acute cholecystitis	Chest pain and diaphoresis	SB	Cholecystectomy	Not reported	No	No
Vloka et al. (1999) [27]	2	Gangrenous GB	Syncope in one case	SB	Cholecystectomy	Not clear	Yes	No
Akyel et al. (2011) [28]	1	Acute calculous cholecystitis	Asymptomatic	Idioventricular rhythm	Conservative	No	No	No
Our case	1	Biliary colic and cholelithiasis	Dizziness	SB	Analgesia	No	No	No

Assessment and workup

Diagnosing CBR requires exclusion of primary cardiac causes. Serial ECGs typically do not show dynamic changes consistent with ischemia, and high-sensitivity troponin levels are usually normal, helping distinguish CBR from myocardial infarction [3,4,25]. Echocardiography is essential for assessing cardiac structure and function, particularly to rule out wall motion abnormalities suggestive of ACS. Ambulatory monitoring may be needed to capture hemodynamically significant bradyarrhythmias, guiding the decision for temporary interventions such as atropine or temporary pacing.

Ultrasonography remains the cornerstone imaging modality for identifying cholelithiasis, gallbladder wall thickening, pericholecystic fluid, and sonographic Murphy’s sign. The most critical diagnostic clue is the temporal association between biliary pain or manipulation and the onset of cardiac symptoms, along with symptom resolution following treatment of the biliary disease [2,6,26].

Management and resolution

Management of CBR involves both acute arrhythmia control and definitive treatment of the underlying biliary pathology. Symptomatic bradycardia can be rapidly reversed with vagolytic agents such as atropine, which may serve both diagnostic and therapeutic purposes [2,4,10]. Analgesia is crucial to remove the nociceptive stimulus triggering the reflex, and in some cases, as demonstrated in our patient, it may be sufficient to reverse bradycardia [6,17]. Emerging evidence suggests that esketamine, an N-methyl-D-aspartate receptor antagonist, may suppress the vagal reflex arc when administered preemptively during surgery, thereby reducing the incidence of CBR [19]. Temporary pacing is indicated for persistent bradyarrhythmia with hemodynamic compromise, providing circulatory support until the reflex abates [6,15,20,22,27,28]. Definitive resolution requires removal of the stimulus, with cholecystectomy being curative; arrhythmias typically resolve postoperatively. As illustrated in our case, conservative management with analgesia and antibiotics can resolve the reflex by reducing inflammation and distension, but surgery remains the definitive preventive measure for recurrence. Permanent pacemakers are rarely required and should only be considered if intrinsic, irreversible sinus node, or atrioventricular node dysfunction persists after treatment of the biliary pathology [22,29].

## Conclusions

This case illustrates a classic presentation of CBR, in which acute gallbladder disease manifested with significant sinus bradycardia that resolved with conservative management. Although rare, CBR is clinically important and can mimic primary cardiac conditions. A high index of suspicion is essential to avoid misdiagnosis and unnecessary cardiac interventions. The cornerstone of management involves recognizing the connection between biliary pathology and cardiac manifestations, providing immediate symptomatic support with analgesics, vagolytic agents, or pacing if needed, and pursuing definitive treatment of the underlying biliary disease. This report reinforces the importance of a holistic diagnostic approach in patients presenting with abdominal pain and concomitant bradyarrhythmia.
